# The Plantain Proteome, a Focus on Allele Specific Proteins Obtained from Plantain Fruits

**DOI:** 10.1002/pmic.201700227

**Published:** 2018-02-23

**Authors:** Nádia A. Campos, Rony Swennen, Sebastien C. Carpentier

**Affiliations:** ^1^ Department of Biosystems KU Leuven Leuven Belgium; ^2^ International Institute of Tropical Agriculture Arusha Tanzania; ^3^ Bioversity International Leuven Belgium; ^4^ SYBIOMA: Facility for SYstems BIOlogy based MAss spectrometry KU Leuven Leuven Belgium

**Keywords:** allele, amino acid polymorphisms, non‐model proteomics, plantain

## Abstract

Proteomics has been applied with great potential to elucidate molecular mechanisms in plants. This is especially valid in the case of non‐model crops of which their genome has not been sequenced yet, or is not well annotated. Plantains are a kind of cooking bananas that are economically very important in Africa, India, and Latin America. The aim of this work was to characterize the fruit proteome of common dessert bananas and plantains and to identify proteins that are only encoded by the plantain genome. We present the first plantain fruit proteome. All data are available via ProteomeXchange with identifier PXD005589. Using our in‐house workflow, we found 37 alleles to be unique for plantain covered by 59 peptides. Although we do not have access (yet) to whole‐genome sequencing data from triploid banana cultivars, we show that proteomics is an easily accessible complementary alternative to detect different allele specific SNPs/SAAPs. These unique alleles might contribute toward the differences in the metabolism between dessert bananas and plantains. This dataset will stimulate further analysis by the scientific community, boost plantain research, and facilitate plantain breeding.

Studies in plant biology through proteomics have increased considerably in the recent years. The main cause is that proteomics provides an insight into the metabolism, and is thus complementary to genomics results.^1^ It is known that the correlation between mRNA and protein at the same moment of extraction is often low.^2^, ^3^ High throughput proteomics for non‐model plants has been used to bypass this problem and to generate more applicable results.^4^, ^5^ Polyploidy and allopolyploidy considerably complicate the proteome analysis of crops. Bananas and plantains are polyploid crops originated from two wild diploid species: *Musa acuminata* (AA), which is highly polymorphous, with spindly plants that grow in clumps, and *Musa balbisiana* (BB), a more homogeneous hardy plant with a massive pseudo‐trunk. There are nowadays diploid, triploid, or tetraploid genome groups.^6^, ^7^ The main genome groups are AA, AB, AAA, AAB, and ABB. Most dessert banana cultivars are AAB or AAA. The Cavendish subgroup, that is sold on the export market^8^ has an AAA genome constitution while plantains are AAB. Plantains are sweet acid starchy bananas with typically long fruits and are mostly consumed after frying or boiling. Plantains are an important staple crop in West and Central Africa, India, and Latin America.^6^ Both dessert bananas and plantains are considered a non‐model crop and the complexity of their genomes makes it challenging to analyze the transcriptome and the proteome.^9^ We used here an easy and reproducible protocol for protein extraction and identification and we present the first proteome of plantain fruits (AAB). We created our own workflow to tackle the difficulties of working with a triploid non‐model species without an available database. The mass spectrometry proteomics data have been deposited at the ProteomeXchange Consortium Partner Repository^10^ via PRIDE^11^ with the dataset identifier PXD005589. These results will stimulate further analysis by the scientific community and will boost plantain research and facilitate breeding.

Plantains fruits and Cavendish fruits were bought in the local supermarket in Leuven, Belgium. Five biological replicates (fruits) of each cultivar were selected based on their phenotypic characteristics and the same green peel color. All fruits were kept separately, cleaned, peeled, their pulp was cut into thin slices and immersed immediately in liquid nitrogen. All ten samples where lyophilized to a water content of 2.5%. After drying, the samples were hermetically sealed and stored at room temperature until the proteomics analysis was performed. Banana tissues are considered difficult for protein extraction due to the presence of many interfering compounds which makes the extraction process more difficult.^12^ Lyophilization provides easier material for manipulation without losses in protein content and is an easy and safe way to transport the samples.^12^ Protein extractions were performed according to the phenol extraction/ammonium acetate precipitation method we published^13^ and adapted for gel free proteomics.^14^

Twenty μg of proteins were digested with trypsin (Trypsin Protease, MS Grade Thermo Scientific) and purified by Pierce C18 Spin Columns (Thermo Scientific). The digested samples (0.5 μg/5 μL) were separated in an Ultimate 3000 (Thermo Scientific) UPLC system and then in a Q Exactive Orbitrap mass spectrometer (Thermo Scientific) as described.^15^ The Q Exactive Orbitrap mass spectrometer (Thermo Scientific, USA) was operated in positive ion mode with a nano spray voltage of 1.5 kV and a source temperature of 250 °C. Proteo Mass LTQ/FT‐Hybrid ESI Pos. Mode Cal Mix (MS CAL5‐1EASUPELCO, Sigma‐Aldrich) was used as an external calibrant and the lock mass 445.12003 as an internal calibrant. The instrument was operated in data‐dependent acquisition (DDA) mode with a survey MS scan at a resolution of 70 000 (fw hm at *m/z* 200) for the mass range of *m/z* 400–1600 for precursor ions, followed by MS/MS scans of the top ten most intense peaks with +2, +3, +4, and +5 charged ions above a threshold ion count of 16 000 at 17 500 resolution using normalized collision energy (NCE) of 25 eV with an isolation window of 3.0 *m/z* and dynamic exclusion of 10 s. All data were acquired with Xcalibur 3.0.63 software (Thermo Scientific). For protein identification, we used MASCOT version 2.2.06 (Matrix Science) against our in house *Musa* A‐B database containing *acuminata* AA proteins (dh PahangV1), the non‐redundant unique *balbisiana* BB proteins (PKW) (http://banana-genome-hub.southgreen.fr/) and the usual contaminants for mass spectrometry (76 220 proteins). The parameters used to search were: parent mass tolerance of 10 PPM, fragment tolerance of 0.02 Da, oxidation of M as variable modification, carbamidomethyl C as fixed modification and up to one missed cleavage was allowed for trypsin. Results from MASCOT were imported to Scaffold version 3.6.5. In Scaffold, the threshold was set to minimum one peptide identified with 95% confidence and the false discovery rate (FDR) was automatically calculated based on default parameters from the software.

Using our *Musa* A‐B database we identified in total 2144 different proteins with 0.2% FDR (Supporting Information, Table 1). Taking into account only the proteins identified in at least two biological replicates reduces this number to 1731, of which 1344 proteins were identified in Cavendish fruits and 1363 in plantain fruits (Supporting Information, Table 1). Esteve et al.^16^ utilized the proteominer beads to identify the proteome of Cavendish fruits. The authors were able to identify 1131 proteins using a cross species approach (Musa EST database and Uniprot Viridiplantae Database). The three most abundant annotation categories were oxidation‐reduction, ATP binding, and nucleotide binding. In our work, we used a merged database derived from two diploid species (AA and BB). We identified and annotated, 4 years later, more proteins due to the availability of more powerful mass spectrometry and more genetic resources. In the category Molecular Function, 525 different gene ontologies could be retrieved. The five most represented ontologies were GO:0016491 oxidoreductase activity (168 proteins); GO:0016787 hydrolase activity (122 proteins); GO:0000166 nucleotide binding (112 proteins); GO:0003824 catalytic activity (103 proteins); and GO:0005524 ATP binding (99 proteins) (Supporting Information, Table 1). The aim of our study was to characterize the proteome of plantain fruits and compare it to Cavendish fruits to identify important allele specific proteins in a cultivar that is not sequenced, plantain. The main contrasting characteristics between plantain and Cavendish are undoubtedly related to unique alleles that can explain together with epigenetic regulations the different phenotypes.^17^ To find allele specific peptides in plantain fruits, we used a basic but very useful principle: spectral counting (Scaffold). Potential plantain allele specific peptides were filtered using the following conditions. Maximum spectral count in Cavendish = 0, which means the peptide was never identified in Cavendish; median spectral count in plantain ≠ 0, being identified at least in three biological replicates. To detect single amino acid polymorphisms (SAAPs) in *acuminata* (A) and *balbisiana* alleles (B), the identified plantain unique peptides were filtered further. Only peptide sequences that were exclusively identified in a B derived protein accession were accepted. Their allelic *acuminata* homolog was searched using the Greenphyl homolog function (http://www.greenphyl.org/cgi-bin/get_homologs.cgi) to determine the SAAP. Only plantain specific proteins where the *acuminata* homolog was successfully identified were accepted (Supporting Information, Table 2). This allowed us to allocate a protein as an A and B allele version. Further annotations of the proteins were retrieved from Uniprot software (http://www.uniprot.org/uploadlists/). Analysis of gene functions from the allelic specific proteins were made through GO enrichment annotations via our in house software (https://labtrop.shinyapps.io/UniGO/).

Following our workflow, we identified 37 interesting loci spread over all 11 chromosomes (Table 1). We appointed 59 peptides as B allele specific and 47 peptides as A allele specific. The introduction of *M. balbisiana* genes is said to be correlated to hardiness, drought tolerance, a changed nutritional value, increased starchiness, and different maturation process.^18^, ^19^, ^20^ To check which pathways are affected by mutations/polymorphisms, we performed a GO annotation for the 37 loci. GO:0004134 (4‐alpha‐glucanotransferase activity) and GO:0004133 (glycogen debranching enzyme activity) are the two most significant GOs for Molecular Function (*p*‐value 3.4e‐06 and 2.0e‐05, respectively) (Supporting Information, Table 3). One single amino acid change can drastically affect the function of proteins.^21^, ^22^, ^23^ Through evolution, mutations in the coding region of a gene are likely to have a different biological function, especially if the mutations occur in the protein domain, since they are generally considered as the basic units of protein folding, evolution, and function.^24^

**Table 1:** Overview of the 59 plantain allele specific peptides and their allelic variant

Uniprot entry and protein annotation	BB accession number^a^	AA accession number^a^	B allele^b^, ^c^	A allele^b^, ^c^, ^d^
M0S0K5 Protein disulfide‐isomerase	KMMuB_chr1_G01477	GSMUA_Achr1T16970_001	AAS**I**LSKNDPPVVLAK	AAS**V**LSKNDPPVVLAK
			EADGI**V**EYLKK	EADGI**I**EYLK
			LHE**V**AENYKGK	LHE**T**AENYKGK
M0S1P5 Uncharacterized protein	KMMuB_chr1_G01832	GSMUA_Achr1T20870_001	AGV**EN**MFGVVGIPVTAVATR	AGV**QH**MFGVVGIPVTAVATR
			ATHITQIPR	No ID
			GYL**A**GTPEELKSALSESFSAR	GYL**V**GTPEELKSALSESFSAR
			LNWLLHFG**Q**PPR	LNWLLHFG**E**PPR
			SHPWVEAISKK	No ID
M0RFU7 D‐3‐phosphoglycerate dehydrogenase	KMMuB_chr10_G29495	GSMUA_Achr10T06200_001	GLGMHVI**S**HDPYAPADR	GLGMHVI**A**HDPYAPADR
M0RKL2 Uncharacterized protein	KMMuB_chr10_G30906	GSMUA_Achr10T22870_001	LVLPGELAK	No ID
M0RPM3 Pectinesterase	KMMuB_chr11_G32183	GSMUA_Achr11T05430_001	SNTNLMFMGDGIGK	No ID
M0RQL6 Uncharacterized protein	KMMuB_chr11_G32507	GSMUA_Achr11T08860_001	IV**Q**DQSVLQDEKR	IV**H**DQSVLQDEKR
M0RS60 Uncharacterized protein	KMMuB_chr11_G33244	GSMUA_Achr11T14300_001	YGVKPDAETLDILNT**I**AR	YGVKPDAETLDILNT**V**AR
M0RTB8 Uncharacterized protein	KMMuB_chr11_G33678	GSMUA_Achr11T18380_001	K**T**IEDLSSSHEK	K**I**IEDLSSSHEK
			SRDLGLDTSTLSK	No ID
M0RTW1 Uncharacterized protein	KMMuB_chr11_G33846	GSMUA_Achr11T20310_001	LV**P**VGYGIK	LV**A**VGYGIK
			T**C**ISGDQISKDDV**K**	T**Y**ISGDQISKDDV**R**
			WY**D**SVFGILA**P**RFPGK	WY**E**SVSGILA**L**RFPGK
M0RUN0 Ubiquitin carboxyl‐terminalhydrolase	KMMuB_chr11_G34086	GSMUA_Achr11T23000_001	F**V**EESFLDRFYK	F**I**EESFLDRFYK
M0S5Q5 Uncharacterized protein	KMMuB_chr2_G03699	GSMUA_Achr2T06640_001	SYITGYQASKDDI**A**VYSALATSPSADYVNVAR	SYITGYQASKDDI**S**VYSALATSPSADYVNVAR
M0S5R0 Uncharacterized protein	KMMuB_chr2_G03706	GSMUA_Achr2T06690_001	LSDAES**MI**ALKDFLNK	LSDAES**LM**ALKDFLNK
M0SA45 4‐alpha‐glucanotransferase	KMMuB_chr2_G05054	GSMUA_Achr2T22040_001	TGDDLPVDYDTRFPSVDPTR	No ID
M0SAT8 Formate dehydrogenase, mitochondrial	KMMuB_chr3_G05265	GSMUA_Achr3T01200_001	AAAE**S**GLTVAEVTGSNVVSVAEDELMR	AAAE**A**GLTVAEVTGSNVVSVAEDELMR
			LKPFNCNLLY**H**DR	LKPFNCNLLY**Y**DR
M0SB08 Sucrose synthase	KMMuB_chr3_G05330	GSMUA_Achr3T01900_001	SVPLAADGEAAFNSAK	No ID
			VVHGIDVFDPKFNIVSPGAD**L**TIYFPYTEK	VVHGIDVFDPKFNIVSPGAD**M**TIYFPYTEK
M0SC42 Pectinesterase	KMMuB_chr3_G05670	GSMUA_Achr3T05740_001	LP**R**PGQINTITAQGR	LP**S**PGQINTITAQGR
M0SCZ5 Uncharacterized protein	KMMuB_chr3_G05965	GSMUA_Achr3T08780_001	S**Y**PVNETNASSSEK	S**D**PVNETNASSSEK
			TIIKDMVLSSER	No ID
M0SIC8 Uncharacterized protein	KMMuB_chr3_G07974	GSMUA_Achr3T27620_001	ALVTELKK	No ID
M0SR05 Malate dehydrogenase	KMMuB_chr4_G10674	GSMUA_Achr4T21920_001	N**A**IIWGNHSSTQYPDV**C**HATVK	N**V**IIWGNHSSTQYPDV**S**HATVK
M0SRM5 Uncharacterized protein	KMMuB_chr4_G10876	GSMUA_Achr4T24140_001	AF**D**SYEAVL**K**DPGVDA**A**YVPLPTSLHLR	AF**G**SYEAVL**E**DPGVDA**V**YVPLPTSLHLR
			AIGLAPN**S**VIVAVGSR	AIGLAPN**A**VIVAVGSR
			HLLLEKPTALCAA**D**LDR	HLLLEKPTALCAA**E**LDR
			WAVAAAE**C**GK	WAVAAAE**R**
M0SVV8 Uncharacterized protein	KMMuB_chr5_G12124	GSMUA_Achr5T05290_001	GYYIQPT**I**FSDVEDKMK	GYYIQPT**V**FSDVEDKMK
M0SWS3 Protein transport Sec61 subunit beta	KMMuB_chr5_G12409	GSMUA_Achr5T08440_001	ARGSSQSQT**T**ASAGGGARPAGAVPR	ARGSSQSQT**A**ASAGGGARPAGAVPR
M0SZK9 Nucleoside diphosphate kinase	KMMuB_chr5_G13683	GSMUA_Achr5T18300_001	GLVGEII**N**RFEK	GLVGEII**S**R
			NVIHGSDSIEGA**S**K	NVIHGSDSIEGA**R**
M0T1H5 Uncharacterized protein	KMMuB_chr5_G14355	GSMUA_Achr5T25000_001	HVT**I**TAFSK	HVT**V**TAFSK
M0SHD1 Importin subunit alpha	KMMuB_chr5_G14553	GSMUA_Achr3T24150_001	SPPIEEVIQAGVVPR	No ID
M0TA69 Uncharacterized protein	KMMuB_chr6_G17518	GSMUA_Achr6T25730_001	EA**S**EKHHHHLF	**E**EA**EEASG**KHHHHLF
M0TB62 Methylthioribose‐1‐phosphate isomerase	KMMuB_chr6_G17823	GSMUA_Achr6T29170_001	LTAFELVHDRIPATLIADSAVA**F**LMK	LTAFELVHDRIPATLIADSAVA**A**LMK
M0TCA9 Uncharacterized protein	KMMuB_chr6_G18191	GSMUA_Achr6T33160_001	IELVPVDLLNRPAWYK**E**K	IELVPVDLLNRPAWYK**D**K
M0TDU3 Uncharacterized protein	KMMuB_chr7_G18687	GSMUA_Achr7T01530_001	ALADQKDEAFF**L**ANAAAQASR	ALADQKDEAFF**S**ANAAAQASR
			CVKPPII**F**GDVSRPK	CVKPPII**Y**GDVSRPK
			EGVKYGAGIGPGVYDIHSPR	No ID
M0TEY6 Uncharacterized protein	KMMuB_chr7_G19042	GSMUA_Achr7T05460_001	ALDEA**A**LVEYIK	ALDEA**S**LVEYIK
			IVGVAHVEDFESISD**E**TKR	IVGVAHVEDFESISD**V**TKR
			YMT**N**LFHDALGFGAAK	YMT**D**LFHDALGFGAAK
M0TIX4 Uncharacterized protein	KMMuB_chr7_G20737	GSMUA_Achr7T19370_001	IATLYSDVLA**A**TILDAEQcKELK	IATLYSDVLTA**T**ILDAEQCKELK
			RLGLVGLGSSSST**V**AAYR	RLGLVGLGSSSST**A**AAYR
			TVD**I**IGFGSGTVVDQK	TVD**V**IGFGSGTVVDQK
M0TJC2 Uncharacterized protein	KMMuB_chr7_G20876	GSMUA_Achr7T20850_001	QFNSIPG**I**MEG**N**AKPDYATCVK	QFNSIPG**L**MEG**T**AKPDYATCVK
M0TRR4 Uncharacterized protein	KMMuB_chr8_G23675	GSMUA_Achr8T19110_001	IMY**A**EDAPDFGAASDGDGDRNMILGR	IMYS**E**DAPDFGAASDGDGDRNMILGR
M0TS86 Uncharacterized protein	KMMuB_chr8_G23861	GSMUA_Achr8T20830_001	MKEIAEAYLGSV**I**K	MKEIAEAYLGSV**V**K
M0U2G0 Uncharacterized protein	KMMuB_chr9_G27509	GSMUA_Achr9T22010_001	GLLSCGTGVGVS**I**FANKFPR	GLLSCGTGVGVS**M**FANKFPR
M0U7I3 Uncharacterized protein	KMMuB_chrUn_random_G35868	GSMUA_AchrUn_randomT08730_001	NKLEDHDELLGADIVQK	No ID
M0UCP7 Uncharacterized protein	KMMuB_chrUn_random_G39488	GSMUA_AchrUn_randomT26890_001	ALQWTIDNLLDIGETL**F**VIHVLRPK	ALQWTIDNLLDIGETL**I**VIHVLRPK

a Chr in the name of the accessions refers to the chromosome number of the locus.

b All peptides have been identified with a probability > 99%.

c SAAPs are indicated in bold.

d No ID: the allelic variant peptide was not confidently identified in our experiment.

Ramu et al.^25^ highlighted some possible deleterious mutations in domesticated cassava using whole genomic screening experiments of wild ancestors and cultivars. Like banana, cassava cultivars are clonally propagated and this genomic screening study suggests that many deleterious mutations have not been crossed out. We expect a similar situation in banana. Advanced whole genomic screening experiments enable the identification and interpretation of mutations at the genome level.^24^, ^25^ Although we do not have access (yet) to whole‐genome sequencing data from triploid banana cultivars, we show that proteomics is an easily accessible complementary alternative to detect the different allele specific SNPs/SAAPs.

To our knowledge, this is the first proteomic investigation in plantain fruits, and the most extensive fruit proteomic study in the genus *Musa*. This public release of the plantain fruit proteome is an important step for plantain varietal selection and breeding.

**Abbreviations**
DDA: data dependent acquisition
EST: expressed sequence tag
FDR: false discovery rate
GO: gene ontology
MS: mass spectrometry
NCE: normalized collision energy
PPM: parts per million
SAAP: single amino acid polymorphism
SNP: single nucleotide polymorphism

## Conflict of Interest

The authors have declared no conflict of interest.

## Supporting information

Supporting Information

Supporting Information

Supporting Information

Supporting Information
