# Supplementary figures and images for: The Plantain Proteome, a Focus on Allele Specific Proteins Obtained from Plantain Fruits
Source: Proteomics. 2018 Feb 23;18(3-4):1700227. doi: 10.1002/pmic.201700227 (PMC6084372; doi:10.1002/pmic.201700227)

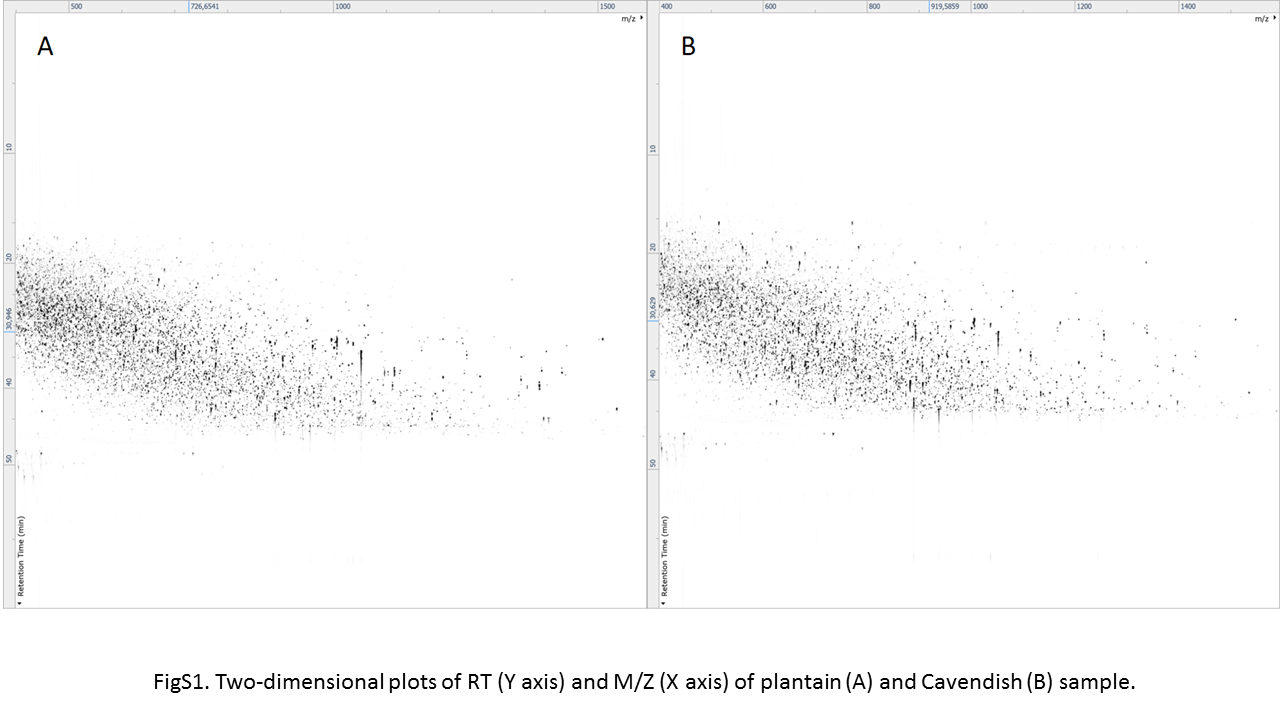

Supplement: Supplementary file 1 — Supporting Information [file PMIC-18-na-s001.tif]
